# Tibial Osteodistraction Angiogenesis for Diabetic Foot Ischemia: A Systematic Review and Meta‐Analysis

**DOI:** 10.1111/wrr.70130

**Published:** 2026-02-12

**Authors:** Arthur Tarricone, Allen Gee, Lee C. Rogers, David C. Lavery, Michael C. Siah, Prakash Krishnan, Dane Wukich, Luke Perry, Matthew Sideman, Lawrence A. Lavery

**Affiliations:** ^1^ Department of Orthopedic Surgery University of Texas Health Science Center San Antonio Texas USA; ^2^ Department of Internal Medicine Mount Sinai Morningside Hospital New York New York USA; ^3^ Statistic Consulting Aurora Colorado USA; ^4^ Department of Plastic Surgery University of Texas Southwestern Medical Center Dallas Texas USA; ^5^ The Zena and Michael A. Wiener Cardiovascular Institute and the Marie‐Josée and Henry R. Kravis Center for Cardiovascular Health, Division of Cardiology, Department of Medicine Icahn School of Medicine at Mount Sinai New York New York USA

**Keywords:** diabetic foot ulcer, ischemic‐non‐healing ulcer, peripheral artery disease, tibial osteotomy distraction angiogenesis, transverse tibial transport

## Abstract

Diabetes‐related lower extremity amputations (LEAs) are a significant global issue, exacerbated by the rising prevalence of diabetes and peripheral artery disease (PAD). Traditional revascularization techniques often fail in patients with severe vascular damage or comorbidities. Tibial Osteodistraction angiogenesis (ODA) represents a novel approach, leveraging angiogenesis to improve perfusion and wound healing. A systematic review was conducted across four databases. Inclusion criteria focused on studies evaluating ODA in diabetic foot ulcers and chronic ischemic wounds. Outcomes analysed included amputation rates, wound healing, mortality, and safety metrics. Eighteen studies (*n* = 3000 treated with ODA) were included, with subjects aged 35–87. Diabetes prevalence was 87.8%. ODA demonstrated high limb salvage rates (92%–100%) and wound healing rates (58%–100%). Pooled analysis revealed amputation rates of 2% and mortality rates of 4%. Angiogenic cytokine levels increased post‐procedure, suggesting enhanced angiogenesis. Complications were minimal, with a 3% pin‐site infection rate and 4% major adverse limb events. ODA offers a promising addition for patients with limb‐threatening ischemia, achieving significant improvements in perfusion, wound healing, and limb salvage. Despite study heterogeneity and limitations, ODA's angiogenic benefits warrant further research through randomised clinical trials to validate its efficacy and broaden clinical adoption.

## 1 Introduction

Diabetes‐related lower extremity amputations (LEAs) are a growing global problem with devastating consequences for both individuals and healthcare systems. The rising prevalence of diabetes, a major risk factor for LEAs, has led to a concerning increase in amputation rates worldwide [1]. In the United States alone, diabetes prevalence surged by 18.6% from 2012 to 2022, resulting in an estimated 154,000 amputations per year [2]. This number continues to rise, disproportionately affecting men and minorities who face a two‐fold higher risk [3, 4, 5]. This disparity highlights the urgent need for targeted interventions and public health initiatives to address the social and economic factors contributing to this inequality. LEAs often result from a combination of factors, primarily peripheral arterial disease (PAD), foot ulceration, and infection [6]. Reduced perfusion impairs wound healing and increases susceptibility to infections, ultimately raising the risk for amputation. Several modifiable risk factors contribute to PAD, including chronic hyperglycemia, unhealthy diet, and smoking [7, 8, 9]. While mitigating these risk factors are important, restoring perfusion is critical to preventing LEAs.

Despite advances in vascular surgery, many people with diabetes and PAD remain at risk for amputation from vascular causes. Up to 20% of people with chronic limb‐threatening ischemia (CLTI) are not candidates for traditional revascularization procedures due to extensive vascular damage, various comorbidities, or poor overall health [10]. Even after successful revascularization, 15%–20% may still require amputation due to ongoing issues like insufficient reperfusion, infection, or other complications. This underscores the limitations of current treatments and emphasizes the need for new therapies to improve perfusion to the feet and promote wound healing.

**FIGURE 1:**
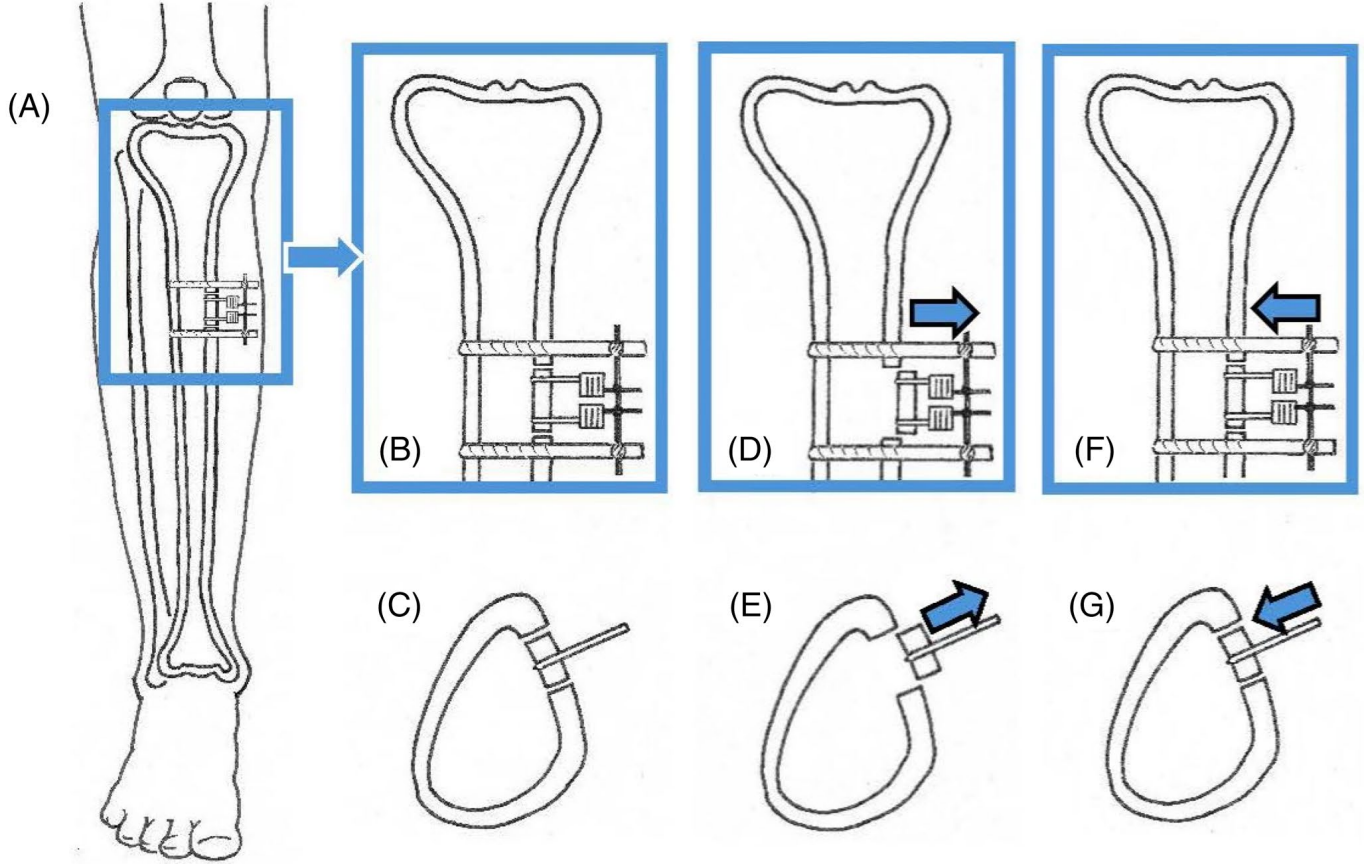
Schematic of the TODA surgery. (A–G) Anterior view of the proximal tibia and cross Section of the tibia to demonstrate location of the osteotomies and distraction of the bone. (A) Shows the anatomic placement of the rectangular osteotomy over the anterior and medial aspect of the proximal tibia. (B) Is an enlarged view showing placement of the pins of the external fixation frame and bone traction needles through the block of bone. (C) Is a cross section of the tibia that shows placement of the bone traction needles. (D) demonstrates distraction of the block of the cortex. The blue arrow shows the direction of bone movement. The bone segment is moved 1 mm a day for 7–14 days. (E) Is a cross section of the bone that shows distraction of the cortex. (F) and (G) shows the movement of the block of bone back to its original position in the tibia.

One such novel approach is tibial osteodistraction osteogenesis (ODA). Garvrill Illizarov popularised the ODA, a technique that takes advantage of endogenous bone regenerative properties to lengthen and reconstruct complex fractures. The process also increases soft tissue angiogenesis. Even though animal and clinical studies have recognised the increase in angiogenesis for several decades, only recently has the procedure been used specifically to increase angiogenesis to treat people with PAD. In 2001, Qu and colleagues described the procedure of transverse tibial bone transport [11]. Tibial osteotomies with distraction of a section of bone have been reported for 20 years to increase angiogenesis and systemic perfusion. We have used the term osteodistraction angiogenesis (ODA) to describe the procedure and its effect more concisely. The procedure involves creating a unicortical window osteotomy on the tibia and gradually distracting the window segment to stimulate angiogenesis, followed by gradually reducing the segment, to release angiogenic cytokines and other healing factors (Figure 1) Early research suggests ODA may improve perfusion, enhance wound healing, prevent major amputations, reduce mortality, and even improve perfusion to the contralateral extremity. This review aims to summarise the available research on ODA (often referred to as TTT in the international literature), including its potential benefits and limitations.

## 2 Methods

**FIGURE 2:**
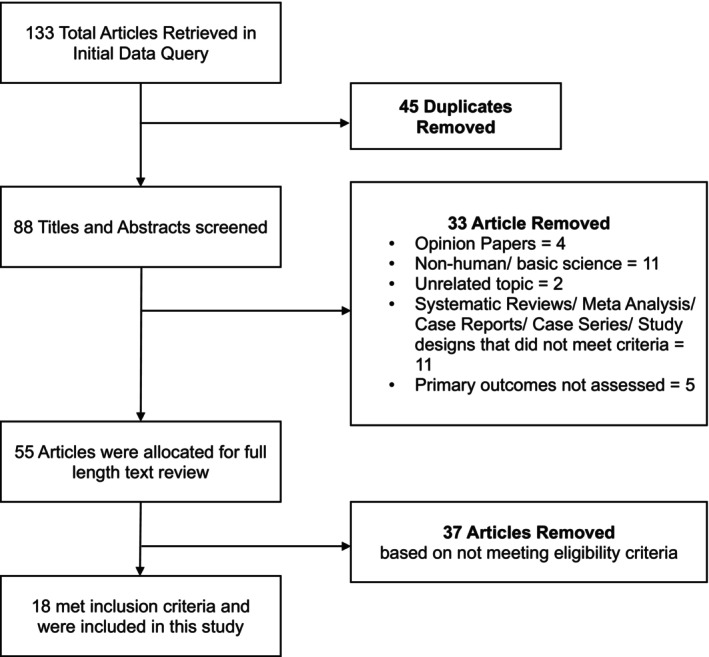
Flow chart of study retrieval.

A literature search was performed on December 12, 2024, across four databases: Pubmed, Embase, Web of Science, and Cochrane libraries. Search terms included TTT, transverse tibial transport, diabetic foot ulcer, diabetic foot infection, DFU, surgical transport, chronic ischemic ulcer, and tibial cortex transverse distraction using the Boolean AND and OR operators (Supplementary 1). A complete flow chart of the study retrieval process can be found in Figure 2. This systematic review and meta‐analysis was registered through the research registry, review number 1954.

All citations were imported to Endnote 20. Duplicates were removed and the remaining citations were screened by their titles and abstracts in accordance with inclusion and exclusion criteria. The inclusion criteria required subjects diagnosed with diabetic foot ulcers (DFUs), non‐healing ulcers from peripheral artery disease, or non‐healing wounds (e.g., wounds secondary to thrombophlebitis) and treated surgically with Osteodistraction Angiogenesis. Studies that were included report outcomes of ODA with mention of amputation, healing, mortality, recurrence, and complication rate, in the English language or have a manuscript in complete translation to the English language, and be of retrospective, prospective, or randomised controlled trial in design. Manuscripts that did not examine the ODA modality, case‐controlled, case series, and other systematic reviews/meta‐analyses were excluded.

After title and abstract screening, the remaining citations were examined in their full length and matched against the inclusion and exclusion criteria. The data from articles determined eligible for this review were then extracted. This included age, BMI, gender distribution, comorbidities, wound classification, imaging methods, type of procedures performed, and general pre and post‐treatment laboratory findings. The primary outcomes assessed in this review included amputation rate [below the ankle (minor) and at/above the ankle (major)], mortality until the end of the follow up period, ulcer/wound recurrence, and healing rate. This study also examined safety outcomes, including pin‐site infections, Major Adverse Limb Events (MALE), Major Adverse Cardiac Events (MACE), and Amputation Free Survival (AFS). MALE was an aggregate of all infections, amputations, or related limb events. MACE was determined by mortality due to myocardial infarctions or stroke. AFS was determined by all patients who survived through the follow up period without experiencing an amputation or dying. The literature review process was carried out by two independent reviewers (LAL and AT), with disagreements resolved by a third independent reviewer (LCR).

All statistical analyses were performed using STATA BE17 and forest plots were generated using R‐Studio. A *p*‐value < 0.05 was considered statistically significant. All categorical variables were reported as *n* (%), while continuous variables were reported as average ± standard deviation. Eggers test and funnel plots were produced to assess publication bias. Heterogeneity was calculated using a *I* ^2^ and a random effects model was used in all analytics.

## 3 Results

A total of 133 articles were retrieved in the initial search. Of these articles, 45 were duplicates and removed from further screening. Titles and abstracts were then reviewed by the investigators and matched based on the inclusion and exclusion criteria. This resulted in the exclusion of an additional 41 articles. An additional 8 articles were then retrieved from external data sources exclusive of the initial query. This resulted in a total of 55 articles to be determined for full‐length analysis. Among these, 18 studies, including 3000 individual subjects treated with ODA, were considered eligible based on this study's inclusion criteria and were included in this systematic review. All studies were conducted in China, between 2019 and 2024. Six studies were comparison in design, and included ODA versus control (debridement, revascularization, negative pressure wound therapy, local or free flaps, and/or skin grafts as indicated), modified ODA (ODA with bone cement) versus traditional ODA, ODA versus a healthy cohort, ODA versus debridement, ODA versus endovascular therapy, and TODA with nose ring drain versus ODA alone [12, 13, 14, 15]. Follow‐up time periods ranged from 3 months to 24 months. A summary of study characteristics is presented in Table 1.

**TABLE 1:** Study characteristics.

Study	Year	Country	Study design	Comparison group	Scoring system	UTSW class	Use of ABIs	Imaging technique
S. Chang 2023	2023	China	Retrospective	N/A	Wagner 3/4	N/A	Pre and Post ABI	CTA and U/S
Y. Chen 2022	2022	China	Prospective	N/A	UTSW Scoring	2C to 3D	Pre ABI	CTA and U/S
Y. Chen 2019	2020	China	Retrospective	TCTD vs. Control	UTSW Scoring	2B to 3D	Pre and Post ABI	CTA and U/S
X. Ding 2022	2022	China	Retrospective	Modified TTT vs. TTT	TEXAS	3D to 4D	Pre and Post ABI	N/A
Z. Fan 2022	2022	China	Retrospective	TTT vs. Normal Healthy	Wagner 3/4	N/A	Pre and Post ABI	CTA and U/S
S. Ou 2022	2022	China	Retrospective	N/A	Wagner 4	N/A	Pre and Post ABI	Angiography
W. Qin 2023	2023	China	Retrospective	N/A	Texas	2B to 3D	No	CTA and U/S
Y. Yuan 2021	2021	China	Retrospective	N/A	Teaxs	2C to 3D	Pre and Post ABI	N/A
L. Zhao 2022	2022	China	Retrospective	N/A	N/A	N/A	No	CTA and U/S
J. Liu 2024	2024	China	Retrospective	N/A	Wagner ≥ 2	N/A	Pre and Post ABI	CTA and U/S
R. Wen 2023	2023	China	Retrospective	TTT vs. debridement	Wagner ≥ 2	N/A	Pre and Post ABI	N/A
D. Xu 2024	2024	China	Retrospective	N/A	Wagner 3/4	N/A	Pre and Post ABI	CTA and U/S
Y. Ding 2024	2024	China	Retrospective	TTT vs. Endovsacular therapy	Rutherford	N/A	N/A	CTA and U/S
Y. Wen 2024	2024	China	Prospective	N/A	Texas	2B to 3D	N/A	N/A
Q. Hua 2020	2020	China	Retrospective	N/A	Wagner ≥ 2	N/A	Pre and Post ABI	N/A
H. Li 2019	2019	China	Retrospective	N/A	Wagner ≥ 2	N/A	N/A	Angiography
J. Yu 2021	2021	China	Retrospective	TTT NRD vs. TTT	Wagner 3/4	N/A	N/A	N/A
D. Zhang 2020	2020	China	Retrospective	N/A	Wagner 3–5	N/A	N/A	Angiography

The age of subjects ranged from 35 to 82 years old. There was a total of 1856 males (61.9%) across all studies. There were many patient comorbidities reported across the studies; however, we only reported those with numerical values for the quantity of each comorbidity. The cumulative sum from the data available showed the number of subjects with diabetes, *n* = 2633 (87.7%), coronary artery disease *n* = 286 (9.5%), active smoking *n* = 349 (11.6%), CKD *n* = 180 (6.0%), peripheral artery disease *n* = 1082 (36.1%), and osteomyelitis *n* = 694 (23.1%) (Table 2).

**TABLE 2:** Subject demographics by study.

Study	*n* Treated with TTT	Age	Male	DM	CAD	ESRD	PAD	Smoker	Osteomyelitis	PAD
S. Chang 2023	13	45–66	9	13	N/A	N/A	N/A	N/A	N/A	No, no mention
Y. Chen 2022	1175	60.4 ± 9.1	752	1052	172	119	871	193	571	871 (81.2%), no mention of Rutherford
Y. Chen 2019	136	61 ± 10	95	134	N/A	20	111	24	74	111 (82%), based on ABI < 0.9
X. Ding 2022	243	68.0–70.4	167	245	N/A	N/A	N/A	N/A	N/A	No mention
Z. Fan 2022	20	42–65	15	N/A	N/A	N/A	N/A	N/A	N/A	No mention
S. Ou 2022	19	67 ± 11.93	9	19	N/A	N/A	N/A	N/A	N/A	No mention
W. Qin 2023	68	64.5 ± 10.5and 64.6 ± 9.8	56	N/A	9	15	45	N/A	N/A	15, based on ABI
Y. Yuan 2021	201	68	107	201	N/A	N/A	N/A	66	N/A	No mention
L. Zhao 2022	20	40 ± 8	20	0	0	N/A	N/A	N/A	N/A	No mention
J. Liu 2024	98	63.6 ± 13	63	68	30	N/A	N/A	N/A	N/A	No mention
R. Wen 2023	35	35–78	18	35	N/A	N/A	N/A	N/A	N/A	No mention
D. Xu 2024	76	64.7 ± 13	49	N/A	37	N/A	N/A	34	N/A	No mention
Y. Ding 2024	55	71.8 ± 11.8	43	25	30	10	55	32	49	Rutherford Grade 5–6
Y. Wen 2024	52	48–82	32	52	N/A	N/A	N/A	N/A	N/A	No mention
Q. Hua 2020	516	68.4	257	516	N/A	N/A	N/A	N/A	N/A	No mention
H. Li 2019	17	46–72	11	17	N/A	N/A	N/A	N/A	0	No mention
J. Yu 2021	60	40–87	44	60	8	16	N/A	N/A	N/A	No mention
D. Zhang 2020	196	45–86	109	196	N/A	N/A	N/A	N/A	N/A	No mention

### 3.1 Classifications

Wounds were graded either by the Wagner Ulcer Classification, University of Texas (UT) Ulcer Classification, or Rutherford classification system. Four studies published results of patients with Wagner Grade 3 or 4 [14, 16, 17, 18], four studies published results with Wagner ≥ Grade 2 [15, 19, 20, 21], one study published results of Wagner Grade 4 only [22], and one study published results of Wagner Grade 3–5 [23]. Only one study reported wounds based on the Rutherford peripheral artery disease classification system, where all subjects in the referenced study had chronic limb threatening ischemia, Rutherford ≥ 5 [24]. The remaining studies used the UT classification system, including two studies with subjects classified as UT Grade 2C to 3D, three studies having classifications UT Grade 2B to 3D, and one study having classifications UT Grade 3D to 4D [12, 13, 25, 26]. One study did not utilise any wound grading system [27].

### 3.2 Perfusion Measurements

Ankle brachial index was used for the diagnosis of peripheral artery disease in all studies. However, only 10 studies reported pre‐ and post‐procedural ABI [12, 13, 14, 15, 16, 19, 22, 25, 26]. The average increase in ABI from pre to post procedure ranged from 0.03 to 0.42, with nine of the 10 studies reporting a significant increase following ODA. Imaging of arteries varied across studies as well. Nine studies used either CT angiogram or ultrasound [12, 14, 16, 17, 19, 24, 25, 27, 28] whereas three used angiography alone [22, 29]. Six studies did not report imaging techniques [13, 15, 18, 20, 26, 29].

### 3.3 Clinical Outcomes

**FIGURE 3:**
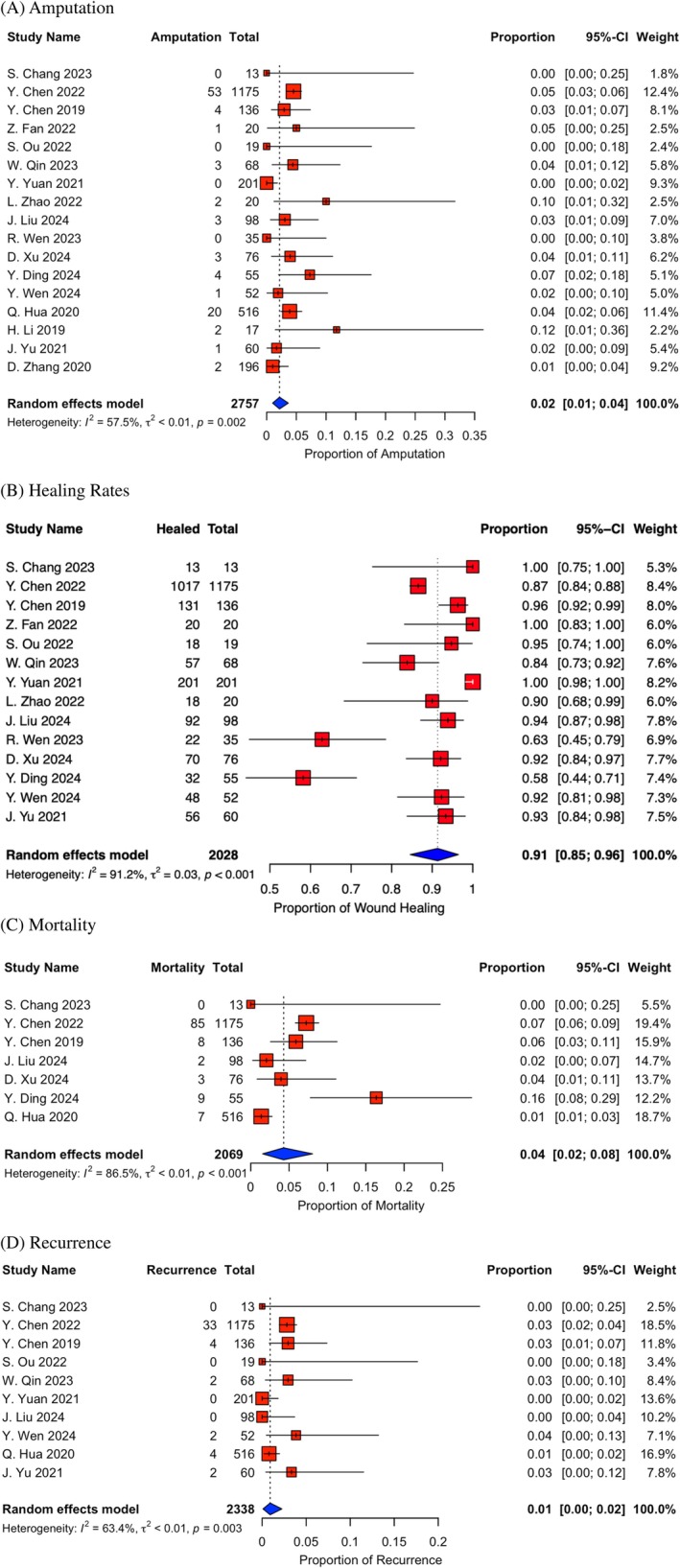
(A) Forest plot of major amputation. (B) Forest plot of wound healing. (C) Forest plot of mortality. (D) Forest plot of ulceration recurrence.

Amputation incidence rates were reported in 17 studies, ranging from 0% to 12% [12, 14, 15, 16, 17, 18, 19, 20, 21, 22, 23, 24, 25, 26, 27, 28, 29]. Five studies reported major amputations only [12, 17, 24, 25, 28], and 12 studies did not have a definition for an amputation [14, 15, 16, 18, 19, 20, 21, 22, 23, 26, 27, 29]. The definition of major amputation was above the ankle. Minor amputation was not defined in any study. In a pooled analysis, the amputation rate was 2% (0.02, CI = 0.01, 0.04, Figure 3a).

The incidence of wound healing was reported in 14 studies, ranging from 58% to 100% [12, 14, 15, 16, 17, 18, 19, 22, 24, 25, 26, 27, 28]. There was also high heterogeneity in the definition of the “wound healing” outcome across the included studies. Six studies defined healing as the complete epithelialization of the ulcer with no drainage of the previous ulcer site and maintaining for at least 2 weeks [19, 22, 25, 26, 27, 28]. In pooled analysis, the incidence of healing was 91% (0.91, CI = 0.85, 0.96, Figure 3b). The remaining studies did not define healing and reported the numeric outcomes only. In addition, the mean healing time was reported in six studies, and averages ranged from 7.1 to 53.2 days [13, 14, 16, 19, 22, 25].

Mortality was reported in seven studies, ranging from 0% to 16% [12, 19, 20, 25]. The definition of mortality in these studies was death during the follow up period. The remaining studies made no mention of mortality in their outcomes. In addition, one study excluded all patients that died in their final analysis [28]. In pooled analysis, the incidence of mortality was 4% (0.04, CI = 0.02, 0.08, Figure 3d).

Ulcer recurrence was reported in 10 studies, ranging from 0% to 4% [12, 16, 18, 19, 20, 22, 25, 26, 28, 29]. Ulcer recurrence was defined as recurrence of the ulcer within the study follow up period. In pooled analysis, the ulcer recurrence rate was 1% (0.01, CI = 0.00, 0.02, Figure 3d).

**FIGURE 4:**
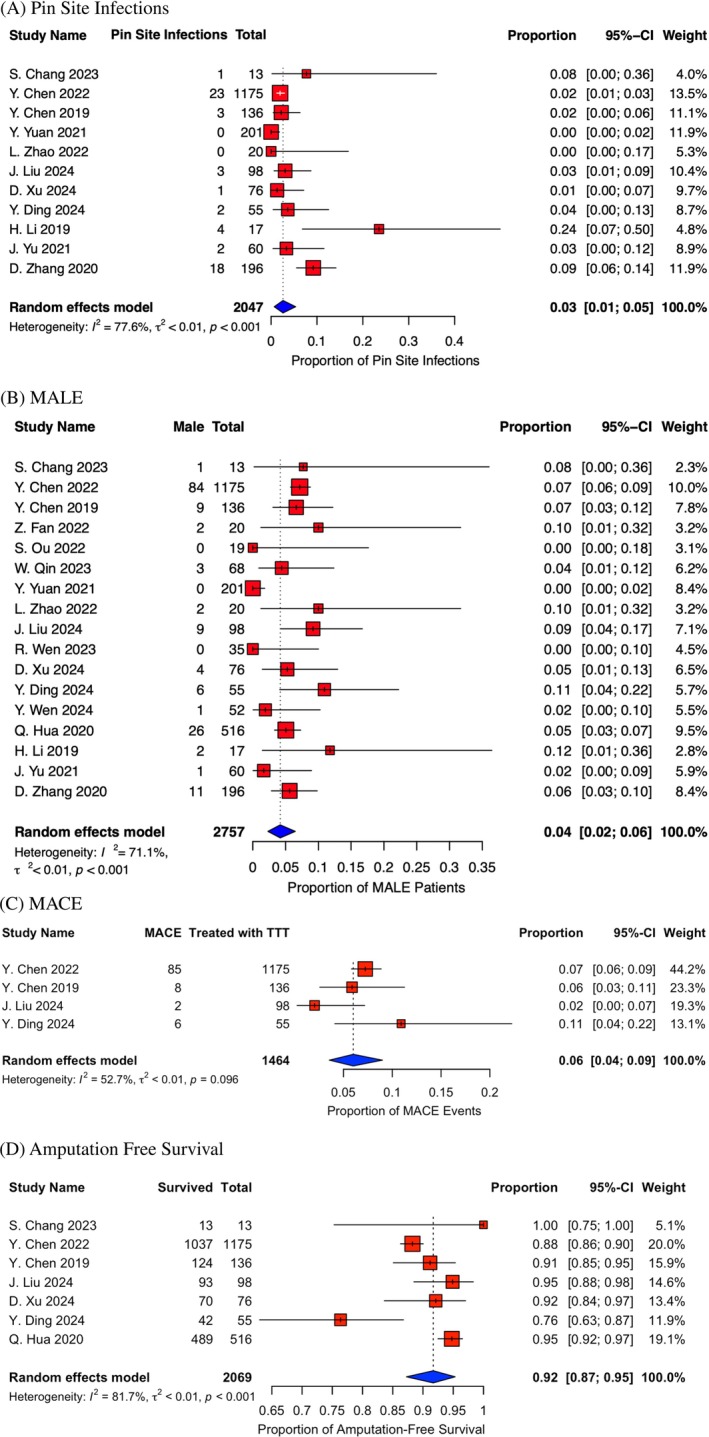
(A) Forest plot of pin site infections. (B) Forest plot of major adverse limb events (MALE). (C) Forest plot of major adverse cardiac event (MACE). (D) Forest plot of amputation free survival (AFS).

Treatment‐related complications or adverse events were uncommon. Pin site infection rate was reported in 11 studies and ranged between 0% and 24%. The overall rate of pin site infection across seven assessed studies was 3% (0.03, CI = 0.01, 0.05, Figure 4a). In addition, the treatment‐related tibial fracture rate was negligible, with three studies reporting no fracture events [16, 22, 26], and five reporting a fracture rate between 0% and 5% [12, 19, 20, 23, 25].

Major Adverse Limb Events (MALE) was determined by an aggregate of limb complications including amputation, fracture, infection of the extremity, or any other complication that involved rehospitalization related to the limb and was reported in 17 studies [12, 14, 15, 16, 17, 18, 19, 20, 21, 22, 23, 24, 25, 26, 27, 28, 29]. Across the studies, the MALE rate among the 2757 subjects was 4% (0.04, CI = 0.02, 0.06, Figure 4b).

Major Adverse Cardiac Events (MACE), defined by myocardial infarction, stroke, or death, were determined in four studies, and found to be 101 out of a total of 1464 subjects, equating to 6.0% (CI = 0.04, 0.09, Figure 4c) [12, 19, 24, 25].

Amputation‐Free Survival was defined as freedom from mortality or major amputation and ranged 76%–100%. The overall proportion of AFS was 92%, and it was calculated from the seven studies that reported this outcome (0.92, CI = 0.87, 0.95, Figure 4d) [12, 16, 19, 25].

### 3.4 Cytokines

The ability of ODA to stimulate angiogenesis is considered the central mechanism of action of the procedure. There were sustained increases in some angiogenic cytokines 3 weeks after the osteodistration procedure was completed. Five studies examined the pre and post‐procedure serum cytokine levels (Table 3) [17, 20, 22, 28, 29]. Xu et al. examined 12 different cytokines in the serum across five different follow up visits for up to 35 days post operation [17]. IL‐6 showed significant elevation at each time point compared with pre‐operative levels. Ou et al. examined the cytokines VEGF, bFGF, EGF, and PDGF at different time points post‐procedure, for up to 35 days as well [22]. By post‐operation day 11, VEGF, bFGF, and EGF were all significantly higher compared with their pre‐procedural baseline. By day 18, PDGF was significantly higher compared with baseline as well. Wen et al. examined three different cytokines: VEGF, Omentin‐1, and Irisin, which were measured at three different timepoints [29]. All three cytokines were significantly elevated at both post‐operative time points compared with their pre‐operative baseline. Qin et al. examined VEGF and SDF‐1 both pre and post operatively, and reported a significant increase in both cytokine levels after the procedure [28]. Hua et al. examined wound tissue expression of Ki‐67, CD31, and VEGF before and after ODA, and reported a higher expression of the three cytokines post procedure [20].

**TABLE 3:** Cytokines.

Cytokines				
S. Ou 2022	Pre‐Operative	Immediate postoperative measurement	Final postoperative measurement	*P* value
VEGF (pg/mL)	71.19 ± 10.29	76.85 ± 8.91	155.01 ± 33.01	< 0.001
bFGF (pg/mL)	47.61 ± 10.91	50.57 ± 10.39	88.99 ± 22.05	< 0.001
EGF (pg/mL)	427.69 ± 169.54	518.36 ± 204.48	744.11 ± 257.72	< 0.001
PDGF (pg/mL)	1932.74 ± 897.21	2334.86 ± 960.27	3123.13 ± 778.73	< 0.001
W. Qin 2023	N/A	N/A	N/A	N/A
VEGF (pg/mL)	~200	~400	~400	< 0.05
SDF‐1 (pg/mL)	~75	~150	~150	< 0.01
Q. Hua 2020	N/A	N/A	N/A	
Ki‐67	N/A	Significantly Elevated		Yes^a^
CD31	N/A	Significantly Elevated		Yes^a^
VEGF	N/A	Significantly Elevated		Yes^a^
D. Xu 2024				
IL‐1 (pg/mL)	~3	~3.5	~3	N/A
IL‐4 (pg/mL)	~1.75	~1.5	~1.5	N/A
IL‐5 (pg/mL)	~0.75	~0.75	~0.75	N/A
IL‐6 (pg/mL)	~19	~35	~19	< 0.05
IL8 (pg/mL)	~39	~40	~40	N/A
TNF‐a (pg/mL)	~2	~1.75	~1.75	N/A
Y. Wen 2024				
VEGF (pg/mL)	75.8 ± 15.5	145.9 ± 30.0	158.6 ± 33.1	< 0.0001
Omentin‐1 (ng/mL)	28.8 ± 8.6	35.5 ± 10.3	42.1 ± 8.3	< 0.0001
Irisin (ng/mL)	14.2 ± 3.9	29.4 ± 4.5	34.2 ± 4.8	< 0.0001

*Note:* ~Indicates an estimated value from interpretation of a chart over time. The exact value for that time period was not reported in the study itself. Table 3 reports the individual cytokines examined in each study. Immediate post‐operative measurement is the first reported value post‐TODA operation. Final post‐operative measurement is the last measurement taken during the study period. N/A indicates no reportable data available.

^a^ Indicates a reported significant change; however, the source text did not provide a *p* value.

### 3.5 Risk of Bias/Publication Bias

A risk of bias assessment was conducted to evaluate the quality of studies, and the Cochrane Risk of Bias 2 (RoB 2) tool was used, assessing domains such as randomization, blinding, missing data, and selective reporting. Overall, there were low to some concern of bias across the studies (Supplementary 2). In addition, this study demonstrates no evidence of publication bias, as indicated by a non‐significant Egger's test (*p* > 0.05) and a symmetrical funnel plot (Supplementary 3).

## 4 Discussion

The results of this study suggest that ODA is a promising treatment option for patients that have wounds with impaired perfusion and are at a high risk of amputation. Across the literature, we found ODA improved healing rates and reduced the risk of major amputation and death. The pooled data show that 91% of patients treated with ODA experienced successful wound healing in 7–53 days. Only 2% of the patients required amputation, and only 4% died. In addition, the findings of this study showed that cytokines were significantly increased following the ODA procedure.

Amputation is one of the most feared complications of diabetes [30]. Below or above‐the‐knee amputation dramatically reduces quality of life [31, 32], reduces the ability to live independently [33, 34], and reduces life expectancy [35, 36]. Lower extremity amputations also have profoundly negative psychological and social implications [37] that lead to depression, anxiety, loss of self‐esteem, and isolation [38]. The emotional and financial burden of an amputee often falls on family members.

Gavriil Ilizarov's pioneering work, arising from an accidental discovery, defined distraction osteogenesis. This technique harnesses the intrinsic regenerative capacity of bone by creating an osteotomy and gradually separating the segments. This controlled separation stimulates the production of new bone tissue, concurrent with increased vascularization in the surrounding soft tissues [39, 40]. Osteodistraction Angiogenesis is based on a similar principle to enhance perfusion in individuals with ischemia. This mechanical distraction initiates a cascade of biochemical signals that promote tissue regeneration, including angiogenesis, modulation of the inflammatory response, and immunomodulatory effects [12, 41, 42]. The studies included in this review demonstrated an increase in perfusion to the foot based on serial arterial Dopplers, transcutaneous oxygen measurements (TCOM), and computer tomography angiography (CTA). Evaluations of pre vs. post‐distraction consistently demonstrate improved perfusion to the foot [12, 16, 25, 28, 29, 43].

The challenges in treating PAD and preventing amputations in individuals with diabetes are further compounded by the complex vascular anatomy of the lower leg and foot. Individuals with diabetes‐related PAD usually have multiple stenoses or occlusions of the three principal arteries below the knee, which makes surgical revascularization technically challenging [44, 45]. Even when successful, re‐occlusion after revascularization is very common [46]. In patients with diabetic foot ischemia, re‐occlusion or re‐stenosis of the treated arteries occurs frequently, limiting long‐term success. The success of revascularization for wound healing is dependent upon restoring adequate perfusion to the affected angiosomes, particularly where there is a wound [44, 47]. If perfusion cannot be optimised, healing is impaired, and the risk of amputation increases. Studies have shown a clear correlation between the number of patent arteries (out of three) supplying the foot and the likelihood of amputation. When two or three arteries are open, the amputation rate is significantly lower compared with situations where only one or no arteries are patent. In many cases, only one or two of the arteries can be successfully treated. Truong and colleagues reported that if two or three vessels are patent to the foot, only 23% of patients will require leg amputation [47]. If there are no vessels or only one vessel that supplies the foot, 61% will require leg amputation. Patients with 0–1 arteries that extend into the foot were 2.5 times (1.4–19.5, *p* = 0.01) more likely to require amputation of the leg. In addition, if there is successful revascularization to an artery but it does not supply blood to the wound's angiosome, the risk of death, poor healing, amputation is 1.8 to 2.6 times higher [44].

In contrast to traditional revascularization techniques that focus on opening specific arteries, the ODA technique offers a unique advantage. ODA stimulates angiogenesis throughout the foot, including areas not directly supplied by the major arteries. This diffuse angiogenic effect may improve perfusion to all areas of the foot, even in the presence of multi‐vessel disease. Furthermore, ODA has been shown to promote angiogenesis in the contralateral limb (untreated side), potentially offering benefits for patients with bilateral PAD. This broader angiogenic effect may explain why ODA has shown promising results in preventing amputations and improving outcomes in patients with limb‐threatening ischemia (sometimes referred to as critical limb ischemia).

There are important limitations involving the existing studies and our analysis. First, there was significant heterogeneity among the studies that were included in terms of study design, patient populations, interventions, and outcome measures. Most of the studies were retrospective, and there were only two prospective cohort studies. There were no randomised clinical trials. Most studies only provided 1 year of follow‐up. None of the studies provided any angiographic classification of PAD before or after surgery. Therefore, it was difficult to understand the severity of PAD within and between studies. These studies commonly represented a cross section of diabetic foot wound complexity based on the Wagner or UT ulcer classification [48]. None of the studies report the frequency or level of foot amputations. All studies reviewed were conducted in China, where there may be differences in the standard of care for wound management and vascular surgery compared with the US and Europe.

While there are promising endpoints that can be extracted from this analysis, study heterogeneity limits the ability to pool data and draw definitive conclusions. The overall quality of the studies that were included in this meta‐analysis was variable, with some studies lacking detailed reporting of methodology and exhibiting potential risks of bias. Most studies were conducted in single centers with limited sample sizes, and all studies were conducted in China without incorporation of any western cohorts; therefore, both factors play the potential in limiting the generalizability of the findings to other populations. Publication bias may also be present, as studies with positive results are more likely to be published. This could lead to an overestimation of the true effect of ODA. This review also was limited in the types of studies, with many retrospective studies, two prospective trials, and no randomised controlled trials. With these considerations in mind, it is important to view the results of this study as hypothesis generating as opposed to concrete evidence for ODA use. Lastly, the number of included studies was relatively small, and the follow‐up periods were generally short. Larger, long‐term studies are needed to confirm these findings and assess the durability of ODA's effects.

## 5 Conclusion

The novel approach of Osteodistraction Angiogenesis offers an option for individuals with limb ischemia who may not be suitable candidates for traditional revascularization procedures, in cases where traditional revascularization fails or in addition to traditional revascularization. While this study found promise in the ability of ODA to heal wounds and prevent major amputations, there are significant limitations in the published studies. Future research should focus on larger, randomised controlled trials to validate the Chinese experience, incorporating Western cohorts and a more diverse patient population. Such studies should include a control arm—such as endovascular revascularization versus ODA—and evaluate long‐term outcomes, including mortality, need for reintervention, and amputation rates.

## Conflicts of Interest

The authors declare no conflicts of interest.

## Supporting information


**Data S1:** Supporting Information.

## Data Availability

The data that support the findings of this study are available in Pubmed at https://pubmed.ncbi.nlm.nih.gov/. These data were derived from the following resources available in the public domain: Pubmed, https://pubmed.ncbi.nlm.nih.gov/.

## References

[1] S. A. Tabish , “Is Diabetes Becoming the Biggest Epidemic of the Twenty‐First Century?,” International Journal of Health Sciences 1, no. 2 (2007): V–VIII. PMC3068646 21475425

[2] S. Neupane , W. J. Florkowski , and C. Dhakal , “Trends and Disparities in Diabetes Prevalence in the United States From 2012 to 2022,” American Journal of Preventive Medicine 67, no. 2 (2024): 299–302. 38648908 10.1016/j.amepre.2024.04.010

[3] R. Saelee , K. M. Bullard , I. A. Hora , et al., “Trends and Inequalities in Diabetes‐Related Complications Among U.S. Adults, 2000‐2020,” Diabetes Care 48, no. 1 (2025): 18–28. 38905540 10.2337/dci24-0022 PMC12425464

[4] J. L. Harding , L. J. Andes , D. B. Rolka , et al., “National and State‐Level Trends in Nontraumatic Lower‐Extremity Amputation Among U.S. Medicare Beneficiaries With Diabetes, 2000‐2017,” Diabetes Care 43, no. 10 (2020): 2453–2459. 32723844 10.2337/dc20-0586 PMC10982954

[5] A. Tarricone , A. Gee , K. De La Mata , et al., “Health Disparities in Nontraumatic Lower Extremity Amputations. A Systematic Review and Meta‐Analysis,” Annals of Vascular Surgery 88 (2023): 410–417. 36210592 10.1016/j.avsg.2022.09.033

[6] A. I. Adler , E. J. Boyko , J. H. Ahroni , and D. G. Smith , “Lower‐Extremity Amputation in Diabetes. The Independent Effects of Peripheral Vascular Disease, Sensory Neuropathy, and Foot Ulcers,” Diabetes Care 22, no. 7 (1999): 1029–1035. 10388962 10.2337/diacare.22.7.1029

[7] M. C. Reyes , M. A. Suludere , A. N. Tarricone , et al., “Residual Diabetic Foot Osteomyelitis After Surgery Leads to Poor Clinical Outcomes: A Systematic Review and Meta‐Analysis,” Wound Repair and Regeneration 32, no. 6 (2024): 872–879. 39376015 10.1111/wrr.13215 PMC11584361

[8] D. M. Alruqayi , J. S. Alsaud , J. M. Alsogaihi , et al., “The Association Between Vitamin B12 Deficiency and Diabetic Foot Ulcer in Type 2 Diabetic Patients in Qassim Province, Saudi Arabia: A Case‐Control Study,” Cureus 16, no. 9 (2024): e68598. 39371761 10.7759/cureus.68598 PMC11450427

[9] P. Sen , T. Demirdal , and B. Emir , “Meta‐Analysis of Risk Factors for Amputation in Diabetic Foot Infections,” Diabetes/Metabolism Research and Reviews 35, no. 7 (2019): e3165. 30953392 10.1002/dmrr.3165

[10] R. Ferraresi , G. Mauri , F. Losurdo , et al., “BAD Transmission and SAD Distribution: A New Scenario for Critical Limb Ischemia,” Journal of Cardiovascular Surgery 59, no. 5 (2018): 655–664. 29786411 10.23736/S0021-9509.18.10572-6

[11] L. Qu , A. Wang , and F. Tang , “The Therapy of Transverse Tibial Bone Transport and Vessel Regeneration Operation on Thromboangitis Obliterans,” Zhonghua Yi Xue Za Zhi 81, no. 10 (2001): 622–624. 11798937

[12] Y. Chen , X. Kuang , J. Zhou , et al., “Proximal Tibial Cortex Transverse Distraction Facilitating Healing and Limb Salvage in Severe and Recalcitrant Diabetic Foot Ulcers,” Clinical Orthopaedics and Related Research 478, no. 4 (2020): 836–851. 31794478 10.1097/CORR.0000000000001075 PMC7282570

[13] X. Ding , Y. Yuan , H. Lu , et al., “Analysis of the Effect of Antibiotic Bone Cement in the Treatment of Diabetic Foot Ulcer Through Tibia Transverse Transport,” Orthopaedic Surgery 14, no. 9 (2022): 2141–2149. 35929648 10.1111/os.13412 PMC9483062

[14] Z. Q. Fan and D. W. Liu , “Impairment Characteristics of Static Balance and Plantar Load Distribution of Patients Undergoing Tibial Cortex Transverse Distraction for Diabetic Foot Ulcers,” Journal of Orthopaedic Surgery and Research 17, no. 1 (2022): 171. 35303911 10.1186/s13018-022-03042-3 PMC8932111

[15] R. Wen , X. Cheng , H. Cao , L. Zhang , F. Luo , and W. Shang , “Transverse Tibial Bone Transfer in the Treatment of Diabetes Foot Ulcer: A Pilot Study,” Diabetes, Metabolic Syndrome and Obesity 16 (2023): 2005–2012. 10.2147/DMSO.S413884 PMC10328219 37427081

[16] S. Chang , F. Zhang , W. Chen , et al., “Outcomes of Integrated Surgical Wound Treatment Mode Based on Tibial Transverse Transport for Diabetic Foot Wound,” Frontiers in Surgery 9 (2022): 1051366. 36726959 10.3389/fsurg.2022.1051366 PMC9885215

[17] D. Xu , C. Bai , R. Hu , et al., “Exploring the Changes in IL‐6 and Related Cytokines in Angiogenesis After Tibial Transverse Transplantation in Diabetic Foot Ulcers,” Orthopaedic Surgery 16, no. 9 (2024): 2181–2190. 39223795 10.1111/os.14221 PMC11572566

[18] J. Yu , Q. Hua , X. Kuang , et al., “Treatment of Severe Diabetic Foot Ulcer Using Tibia Transverse Transport Combined With Nose Ring Drain,” Zhongguo Xiu Fu Chong Jian Wai Ke Za Zhi 35, no. 8 (2021): 984–988. 34387426 10.7507/1002-1892.202103039 PMC8404002

[19] J. Liu , X. Yao , Z. Xu , et al., “Modified Tibial Cortex Transverse Transport for Diabetic Foot Ulcers With Wagner Grade ≥ II: A Study of 98 Patients,” Front Endocrinol (Lausanne) 15 (2024): 1334414. 38318295 10.3389/fendo.2024.1334414 PMC10841573

[20] Q. Hua , S. Qin , X. Kuang , Y. Chen , L. Qu , and J. Zhao , “Treatment Experiences of 516 Cases of Diabetic Foot Treated With Tibial Transverse Transport,” Zhongguo Xiu Fu Chong Jian Wai Ke Za Zhi 34, no. 8 (2020): 959–963. 32794661 10.7507/1002-1892.202003099 PMC8171913

[21] H. Li , J. You , C. Liu , and Y. Ma , “Effectiveness of Transverse Tibial Bone Transport in Treatment of Diabetic Foot Ulcer,” Zhongguo Xiu Fu Chong Jian Wai Ke Za Zhi 33, no. 1 (2019): 23–27. 30644256 10.7507/1002-1892.201807143 PMC8337247

[22] S. Ou , C. Xu , Y. Yang , et al., “Transverse Tibial Bone Transport Enhances Distraction Osteogenesis and Vascularization in the Treatment of Diabetic Foot,” Orthopaedic Surgery 14, no. 9 (2022): 2170–2179. 35946439 10.1111/os.13416 PMC9483085

[23] D. Zhang , J. Huang , B. Shi , and B. Chen , “Analysis of Complications in Diabetic Foot Treated With Tibial Transverse Transport,” Zhongguo Xiu Fu Chong Jian Wai Ke Za Zhi 34, no. 8 (2020): 985–989. 32794666 10.7507/1002-1892.202003114 PMC8171903

[24] Y. Ding , D. Yu , H. Huang , et al., “Combining Tibial Cortex Transverse Transport (TTT) and Endovascular Therapy (EVT) for Limb Salvage in Chronic Limb‐Threatening Ischemia,” Orthopaedic Surgery 16, no. 9 (2024): 2132–2139. 39243174 10.1111/os.14222 PMC11572561

[25] Y. Chen , X. Ding , Y. Zhu , et al., “Effect of Tibial Cortex Transverse Transport in Patients With Recalcitrant Diabetic Foot Ulcers: A Prospective Multicenter Cohort Study,” Journal of Orthopaedic Translation 36 (2022): 194–204. 36263383 10.1016/j.jot.2022.09.002 PMC9576490

[26] Y. Yuan , X. Ding , Z. Jing , et al., “Modified Tibial Transverse Transport Technique for the Treatment of Ischemic Diabetic Foot Ulcer in Patients With Type 2 Diabetes,” Journal of Orthopaedic Translation 29 (2021): 100–105. 34136348 10.1016/j.jot.2021.04.006 PMC8165491

[27] L. Zhao , Y. Lei , M. Pang , and Z. Wei , “An Improved Bone Transport Surgical Method for Treating Chronic Ischemic Ulcers (Thromboangiitis Obliterans),” Frontiers in Surgery 9 (2022): 859201. 36061060 10.3389/fsurg.2022.859201 PMC9437542

[28] W. Qin , X. Nie , H. Su , et al., “Efficacy and Safety of Unilateral Tibial Cortex Transverse Transport on Bilateral Diabetic Foot Ulcers: A Propensity Score Matching Study,” Journal of Orthopaedic Translation 42 (2023): 137–146. 37736148 10.1016/j.jot.2023.08.002 PMC10509564

[29] Y. Wen , L. Chen , J. Lan , and L. Li , “Efficacy of Tibial Cortex Transverse Transport in Treating Diabetic Foot Ulcer and Its Effect on Serum Omentin‐1 and Irisin Levels,” Diabetology and Metabolic Syndrome 16, no. 1 (2024): 154. 38982536 10.1186/s13098-024-01400-1 PMC11232319

[30] D. K. Wukich , K. M. Raspovic , D. C. Jupiter , et al., “Amputation and Infection Are the Greatest Fears in Patients With Diabetes Foot Complications,” Journal of Diabetes and Its Complications 36, no. 7 (2022): 108222. 35717355 10.1016/j.jdiacomp.2022.108222

[31] M. J. Johnson , D. K. Wukich , P. A. Nakonezny , et al., “The Impact of Hospitalization for Diabetic Foot Infection on Health‐Related Quality of Life: Utilizing PROMIS,” Journal of Foot and Ankle Surgery 61, no. 2 (2022): 227–232. 10.1053/j.jfas.2021.07.011 34389216

[32] E. J. Peters , M. R. Childs , R. P. Wunderlich , L. B. Harkless , D. G. Armstrong , and L. A. Lavery , “Functional Status of Persons With Diabetes‐Related Lower‐Extremity Amputations,” Diabetes Care 24, no. 10 (2001): 1799–1804. 11574445 10.2337/diacare.24.10.1799

[33] T. Schoppen , A. Boonstra , J. W. Groothoff , J. de Vries , L. N. Goeken , and W. H. Eisma , “Physical, Mental, and Social Predictors of Functional Outcome in Unilateral Lower‐Limb Amputees,” Archives of Physical Medicine and Rehabilitation 84, no. 6 (2003): 803–811. 12808530 10.1016/s0003-9993(02)04952-3

[34] E. Farber , M. Zhu , T. McNamara , T. W. Cheng , A. Alonso , and J. J. Siracuse , “Patients Experience Significant Long‐Term Social and Health Challenges After Major Lower Extremity Amputation,” Annals of Vascular Surgery 109 (2024): 291–296. 39069122 10.1016/j.avsg.2024.07.087

[35] L. A. Lavery , N. A. Hunt , A. Ndip , D. C. Lavery , W. Van Houtum , and A. J. Boulton , “Impact of Chronic Kidney Disease on Survival After Amputation in Individuals With Diabetes,” Diabetes Care 33, no. 11 (2010): 2365–2369. 20739688 10.2337/dc10-1213 PMC2963496

[36] D. K. Wukich , J. Ahn , K. M. Raspovic , F. A. Gottschalk , J. La Fontaine , and L. A. Lavery , “Comparison of Transtibial Amputations in Diabetic Patients With and Without End‐Stage Renal Disease,” Foot & Ankle International 38, no. 4 (2017): 388–396. 28103735 10.1177/1071100716688073

[37] A. C. Rosca , C. C. Baciu , V. Burtaverde , and A. Mateizer , “Psychological Consequences in Patients With Amputation of a Limb. An Interpretative‐Phenomenological Analysis,” Frontiers in Psychology 12 (2021): 537493. 34122200 10.3389/fpsyg.2021.537493 PMC8189153

[38] E. Dawes , L. L. Hewitt , V. V. Bliokas , and V. J. Wilson , “A Systematic Review of Cognitive Functioning and Its Relationship to Outcomes Following Amputation Secondary to Vascular Etiology,” International Journal of Lower Extremity Wounds 24 (2023): 1156269. 10.1177/15347346231156269 36760137

[39] G. A. Ilizarov , “The Tension‐Stress Effect on the Genesis and Growth of Tissues. Part I. The Influence of Stability of Fixation and Soft‐Tissue Preservation,” Clinical Orthopaedics and Related Research 238 (1989): 249–281. 2910611

[40] W. Hu , Z. Guo , W. Tang , and J. Long , “Mechanoresponsive Regulation of Tissue Regeneration During Distraction Osteogenesis,” FASEB Journal 38, no. 18 (2024): e70056. 39282872 10.1096/fj.202401303R

[41] J. Compton , A. Fragomen , and S. R. Rozbruch , “Skeletal Repair in Distraction Osteogenesis: Mechanisms and Enhancements,” JBJS Reviews 3, no. 8 (2015): 1–12. 10.2106/JBJS.RVW.N.00107 27490473

[42] S. Weiss , R. Baumgart , M. Jochum , C. J. Strasburger , and M. Bidlingmaier , “Systemic Regulation of Distraction Osteogenesis: A Cascade of Biochemical Factors,” Journal of Bone and Mineral Research 17, no. 7 (2002): 1280–1289. 12096842 10.1359/jbmr.2002.17.7.1280

[43] Z. Q. Fan , Z. H. Yu , J. Z. Zheng , B. F. Yu , and D. W. Liu , “Tibial Cortex Transverse Distraction in Treating Diabetic Foot Ulcers: What Are We Concerned About?,” Journal of International Medical Research 48, no. 9 (2020): 954697. 10.1177/0300060520954697 PMC7509750 32951489

[44] A. Tarricone , A. Gee , K. de la Mata , et al., “Outcomes for Patients With Chronic Limb‐Threatening Ischemia After Direct and Indirect Endovascular and Surgical Revascularization: A Meta‐Analysis and Systematic Review,” Journal of Endovascular Therapy 33 (2024): 248524. 10.1177/15266028241248524 PMC12804421 38687701

[45] J. Chung , J. G. Modrall , M. Knowles , et al., “Arteriographic Patterns of Atherosclerosis and the Association Between Diabetes Mellitus and Ethnicity in Chronic Critical Limb Ischemia,” Annals of Vascular Surgery 40 (2017): 198–205. 27908824 10.1016/j.avsg.2016.11.003

[46] M. Meloni , V. Izzo , L. Giurato , et al., “Recurrence of Critical Limb Ischemia After Endovascular Intervention in Patients With Diabetic Foot Ulcers,” Advances in Wound Care (New Rochelle) 7, no. 6 (2018): 171–176. 10.1089/wound.2017.0778 PMC5994148 29892493

[47] D. H. Truong , A. K. Ngoo , S. Tsai , A. K. Yang , D. K. Wukich , and L. A. Lavery , “Success of Transmetatarsal Amputation for Limb Salvage in Patients With Peripheral Artery Disease,” International Wound Journal 21, no. 1 (2024): e14360. 37622404 10.1111/iwj.14360 PMC10781589

[48] D. G. Armstrong , L. A. Lavery , and L. B. Harkless , “Validation of a Diabetic Wound Classification System. The Contribution of Depth, Infection, and Ischemia to Risk of Amputation,” Diabetes Care 21, no. 5 (1998): 855–859. 9589255 10.2337/diacare.21.5.855

