# Supplementary material for: Tibial Osteodistraction Angiogenesis for Diabetic Foot Ischemia: A Systematic Review and Meta‐Analysis
Source: Wound Repair Regen. 2026 Feb 12;34(1):e70130. doi: 10.1111/wrr.70130 (PMC12895301; doi:10.1111/wrr.70130)

**Supplementary 1: Search Strategy**

**PubMed Search Strategy**

Search: (((TTT) OR (Transverse Tibial Transport))) AND ((Diabetic Foot Ulcer) OR (Diabetic Foot Infection) OR DFU))

("TTT"[All Fields] OR (("transversal"[All Fields] OR "transversally"[All Fields] OR "transversals"[All Fields] OR "transverse"[All Fields] OR "transversed"[All Fields] OR "transversely"[All Fields] OR "transverses"[All Fields] OR "transversing"[All Fields]) AND ("tibia"[MeSH Terms] OR "tibia"[All Fields] OR "tibial"[All Fields] OR "tibialization"[All Fields] OR "tibially"[All Fields] OR "tibials"[All Fields]) AND ("biological transport"[MeSH Terms] OR ("biological"[All Fields] AND "transport"[All Fields]) OR "biological transport"[All Fields] OR "transport"[All Fields] OR "membrane transport proteins"[MeSH Terms] OR ("membrane"[All Fields] AND "transport"[All Fields] AND "proteins"[All Fields]) OR "membrane transport proteins"[All Fields] OR "transporter"[All Fields] OR "transporters"[All Fields] OR "transportable"[All Fields] OR "transportation"[MeSH Terms] OR "transportation"[All Fields] OR "transportations"[All Fields] OR "transported"[All Fields] OR "transporter s"[All Fields] OR "transporting"[All Fields] OR "transports"[All Fields]))) AND ("diabetic foot"[MeSH Terms] OR ("diabetic"[All Fields] AND "foot"[All Fields]) OR "diabetic foot"[All Fields] OR ("diabetic"[All Fields] AND "foot"[All Fields] AND "ulcer"[All Fields]) OR "diabetic foot ulcer"[All Fields] OR (("diabetic foot"[MeSH Terms] OR ("diabetic"[All Fields] AND "foot"[All Fields]) OR "diabetic foot"[All Fields]) AND ("infect"[All Fields] OR "infectability"[All Fields] OR "infectable"[All Fields] OR "infectant"[All Fields] OR "infectants"[All Fields] OR "infected"[All Fields] OR "infecteds"[All Fields] OR "infectibility"[All Fields] OR "infectible"[All Fields] OR "infecting"[All Fields] OR "infection s"[All Fields] OR "infections"[MeSH Terms] OR "infections"[All Fields] OR "infection"[All Fields] OR "infective"[All Fields] OR "infectiveness"[All Fields] OR "infectives"[All Fields] OR "infectivities"[All Fields] OR "infects"[All Fields] OR "pathogenicity"[MeSH Subheading] OR "pathogenicity"[All Fields] OR "infectivity"[All Fields])) OR "DFU"[All Fields])

Results 61

Search: (Surgical transport) AND (chronic ischemic ulcer)

("surgical procedures, operative"[MeSH Terms] OR ("surgical"[All Fields] AND "procedures"[All Fields] AND "operative"[All Fields]) OR "operative surgical procedures"[All Fields] OR "surgical"[All Fields] OR "surgically"[All Fields] OR "surgicals"[All Fields]) AND ("biological transport"[MeSH Terms] OR ("biological"[All Fields] AND "transport"[All Fields]) OR "biological transport"[All Fields] OR "transport"[All Fields] OR "membrane transport proteins"[MeSH Terms] OR ("membrane"[All Fields] AND "transport"[All Fields] AND "proteins"[All Fields]) OR "membrane transport proteins"[All Fields] OR "transporter"[All Fields] OR "transporters"[All Fields] OR "transportable"[All Fields] OR "transportation"[MeSH Terms] OR "transportation"[All Fields] OR "transportations"[All Fields] OR "transported"[All Fields] OR "transporter s"[All Fields] OR "transporting"[All Fields] OR "transports"[All Fields]) AND (("chronic"[All Fields] OR "chronical"[All Fields] OR "chronically"[All Fields] OR "chronicities"[All Fields] OR "chronicity"[All Fields] OR "chronicization"[All Fields] OR "chronics"[All Fields]) AND ("ischaemics"[All Fields] OR "ischemia"[MeSH Terms] OR "ischemia"[All Fields] OR "ischaemic"[All Fields] OR "ischemic"[All Fields] OR "ischemical"[All Fields] OR "ischemically"[All Fields] OR "ischemics"[All Fields] OR "ischemized"[All Fields]) AND ("ulcer"[MeSH Terms] OR "ulcer"[All Fields] OR "ulcerate"[All Fields] OR "ulcerated"[All Fields] OR "ulcerates"[All Fields] OR "ulcerating"[All Fields] OR "ulceration"[All Fields] OR "ulcerations"[All Fields] OR "ulcerative"[All Fields] OR "ulcers"[All Fields] OR "ulcer s"[All Fields] OR "ulcerous"[All Fields]))

Results 9

Search: (Tibial Cortex Transverse Distraction) AND (diabetic foot ulcer)

("tibia"[MeSH Terms] OR "tibia"[All Fields] OR "tibial"[All Fields] OR "tibialization"[All Fields] OR "tibially"[All Fields] OR "tibials"[All Fields]) AND ("cortex"[All Fields] OR "cortex s"[All Fields] OR "cortexes"[All Fields]) AND ("transversal"[All Fields] OR "transversally"[All Fields] OR "transversals"[All Fields] OR "transverse"[All Fields] OR "transversed"[All Fields] OR "transversely"[All Fields] OR "transverses"[All Fields] OR "transversing"[All Fields]) AND ("distract"[All Fields] OR "distractability"[All Fields] OR "distractable"[All Fields] OR "distracted"[All Fields] OR "distracter"[All Fields] OR "distracters"[All Fields] OR "distractibility"[All Fields] OR "distractible"[All Fields] OR "distracting"[All Fields] OR "distraction"[All Fields] OR "distractional"[All Fields] OR "distractions"[All Fields] OR "distractive"[All Fields] OR "distracts"[All Fields]) AND ("diabetic foot"[MeSH Terms] OR ("diabetic"[All Fields] AND "foot"[All Fields]) OR "diabetic foot"[All Fields] OR ("diabetic"[All Fields] AND "foot"[All Fields] AND "ulcer"[All Fields]) OR "diabetic foot ulcer"[All Fields])

Results 15

**Embase Search Strategy**

| Search Number | Search Term | Results |
| --- | --- | --- |
| 1 | Diabetic Foot Ulcer | 11,279 |
| 2 | DFU | 3,061 |
| 3 | TTT | 4,202 |
| 4 | Tibial Transverse Transport | 82 |
| 5 | 1 AND 3 | 30 |
| 6 | 1 AND 4 | 44 |
| 7 | 2 AND 3 | 15 |
| 8 | 2 AND 4 | 16 |

**Cochrane Reviews:**

- 0 Articles found in database

**Web of Science:**

- 15 Results

Supplementary 2: Risk of Bias


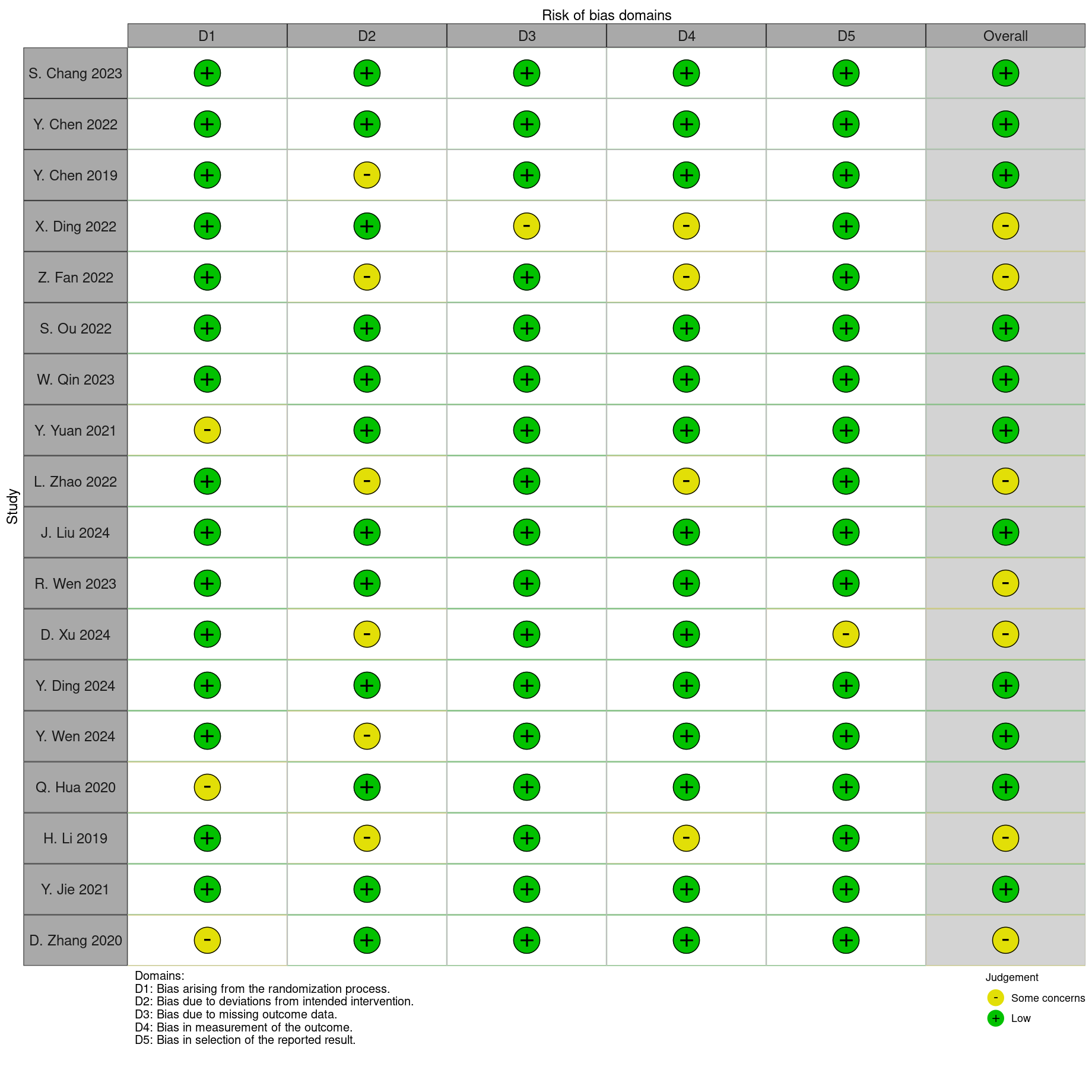


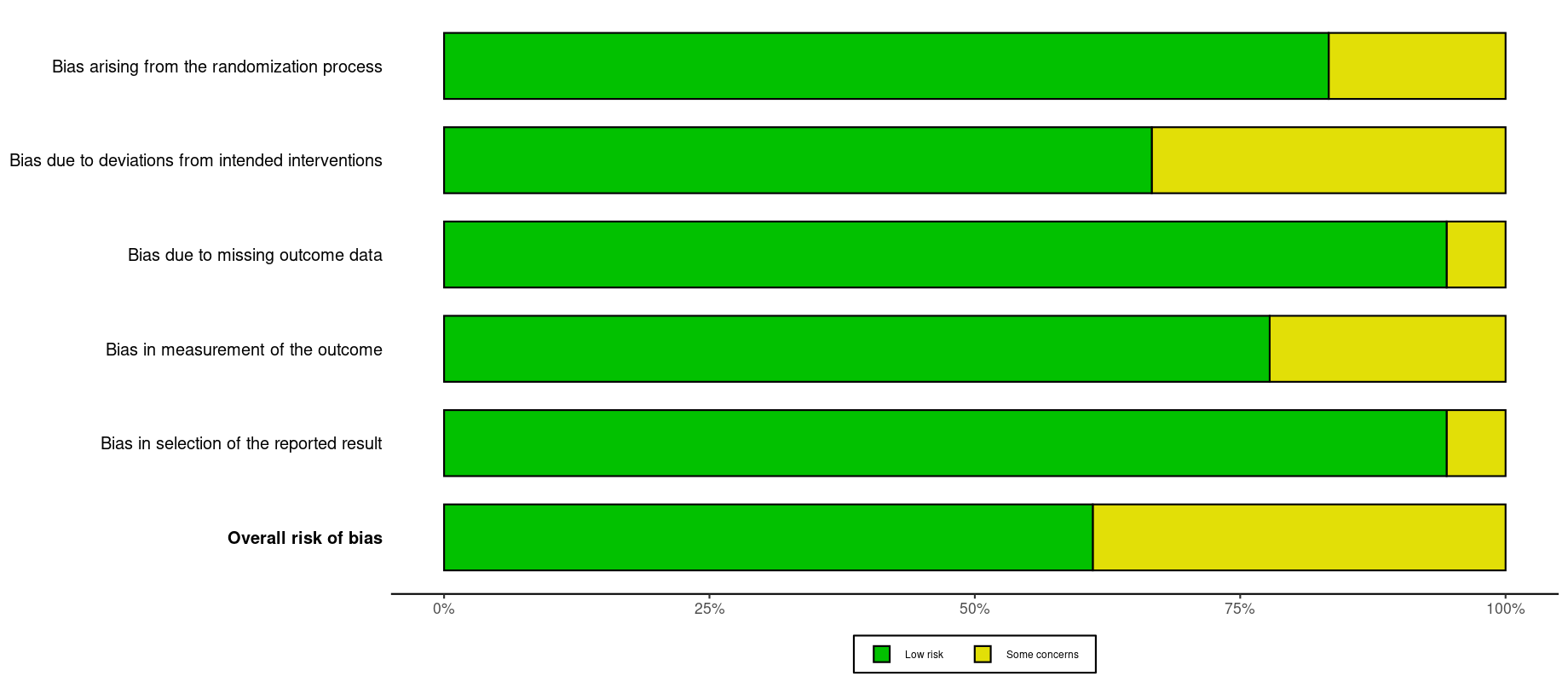


Supplementary 3: Eggers and Funnel Plot


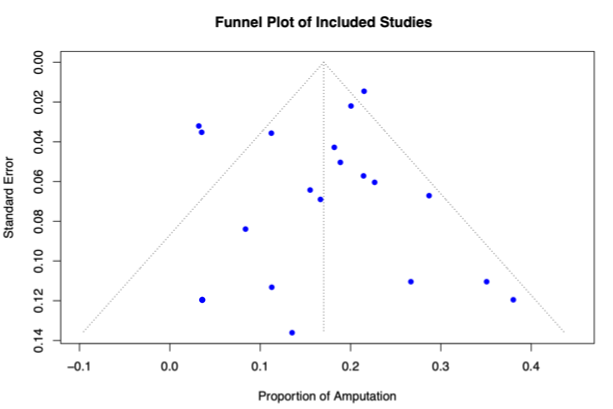

Supplement: Supplementary file 1 — Data S1: Supporting Information. [file WRR-34-0-s001.docx]
